# Analysis of Time-Resolved Gene Expression Measurements across Individuals

**DOI:** 10.1371/journal.pone.0082340

**Published:** 2013-12-09

**Authors:** Laura L. Elo, Benno Schwikowski

**Affiliations:** 1 Department of Mathematics and Statistics, University of Turku, Turku, Finland; 2 Turku Centre for Biotechnology, University of Turku and Åbo Akademi University, Turku, Finland; 3 Systems Biology Lab, Department of Genomes and Genetics, Institut Pasteur, Paris, France; Michigan State University, United States of America

## Abstract

Genetic and environmental determinants of altered cellular function, disease state, and drug response are increasingly studied using time-resolved transcriptomic profiles. While it is widely acknowledged that the rate of biological processes may vary between individuals, data analysis approaches that go beyond evaluating differential expression of single genes have so far not taken this variability into account. To this end, we introduce here a robust multi-gene data analysis approach and evaluate it in a biomarker discovery scenario across four publicly available datasets. In our evaluation, existing methods perform surprisingly poorly on time-resolved data; only the approach taking the variability into account yields reproducible and biologically plausible results. Our results indicate the need to capture gene expression between potentially heterogeneous individuals at multiple time points, and highlight the importance of robust data analysis in the presence of heterogeneous gene expression responses.

## Introduction

Gene expression is regarded as an important molecular phenotype reflecting the genetic and environmental determinants of disease, drug response, and altered cellular function [1]–[6]. In the simplest case, a higher-level phenotype of interest is associated with the abundance of a single gene transcript measured at a given point in time, which may be then identified using a transcriptome measurement at that time in the process. A problem in the case of dynamic biological processes, such as cancer or infection, is the choice of a suitable time point. One reason is that the start of the process is in many cases unknown, and may differ across individuals. Another reason is that the speed of the process may vary between, for instance in individuals of different genetic or epigenetic background [7], [8].

Spurred by the ongoing increase in the capacity of transcriptomic technology, these problems can be addressed by acquiring multiple time points for each sample, and the importance of dynamics of gene expression for the purpose of selecting biomarkers has been recognized [9]. The statistical concept of differential expression has been extended to time series [10], and methods to correct for different speeds of transcriptomic response between individuals in single genes have been developed [11]. Besides their focus on single genes, a major limitation is the common application of these methods to a statistically low number of time points [11], [12].

The model of a single transcript as an indicator of disease state is attractive, but possibly unrealistic [13]. Therefore, the concept of using *signatures* composed of multiple transcripts is gaining traction [14]–[16]. One popular paradigm is the idea of co-regulation and resulting correlated expression of a group of functionally related genes over experimental conditions. The absence of such co-expression has been found to be associated with higher-level phenotypes (e.g., disease state), and the corresponding genes have been used as biomarkers, typically in non-time series data [17].

**Figure 1:**
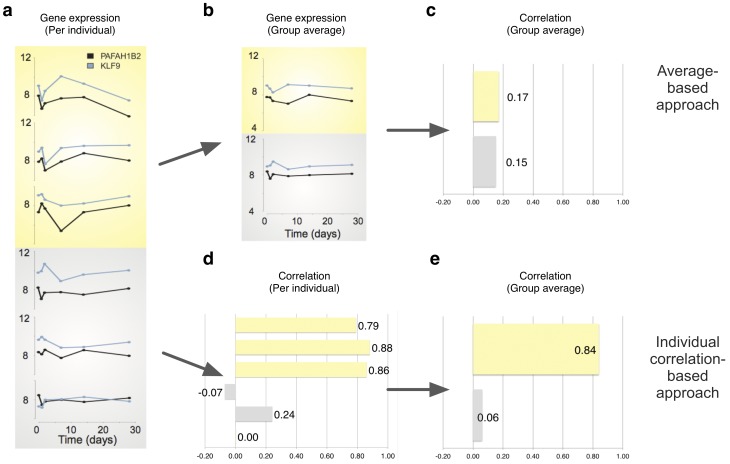
Schematic illustration of two approaches to differential co-expression analysis between groups of individuals. Data are from three responders (yellow) and three nonresponders (grey) to interferon/ribavirin therapy for Hepatitis C infection (genes and individuals selected from larger sets) [25]. **(a)** Gene expression profile for each individual. The responder profiles (yellow) are consistent with the hypothesis of a series of qualitative changes (down–up–down) occurring within each individual, but at different time scales when compared between individuals (the third sequence not being entirely observed). Nonresponder profiles (grey) do not appear particularly correlated. **(b)** Average expression profiles of logarithmic intensities when averaged over the individuals in each group. **(c)** Correlation between average expression profiles. As the average responder time courses in **(b)** do not reflect the strong correlation in the individuals, correlation between average expression profiles appears no stronger than in nonresponders. **(d)** Pairwise correlation coefficient of expression profiles within each individual. **(e)** Average correlation values within each group. Pairwise correlations in responders are significantly higher than in nonresponders, leading to a large difference between average correlation between responders and nonresponders. In this illustration, all correlations are Pearson correlations; averages are arithmetic averages.

One of the first studies to perform differential co-expression analysis on grouped time-series data between two different cell lines was a study by Remondini et al [18]. For each gene, they first determined a single average time series in both groups by averaging the expression values across the replicate sample series. In a second step, for each gene pair, time series correlations were then calculated separately for each group and differential co-expression between the groups was defined as the difference between these correlations (see **Fig. 1** upper path for an illustration). As a final step, all pairwise differential correlation values involving a given gene can be aggregated to produce a ranked list of genes for further study.

The above approach, the now-common weighted gene co-expression network analysis (WGCNA) [19]–[21], and a more recently introduced variant called Differential Co-expression Profile analysis (DCp) [22] are straightforward to apply to multiple individuals in a group after averaging, and various implementations are readily available. While this remains a current *de facto* strategy for the analysis of time-series data [23], [24], we argue here that the initial group averaging step, which makes grouped time-series data accessible to these methods, can be highly problematic in the case of heterogeneity in the transcriptomic response between individuals, because it may lose a large part of the initial correlation. The same holds true for most differential expression approaches applied to time series data, which by default typically ignore the time heterogeneity between the sample series. **Figure 1** illustrates this weakness of the ‘group average’-based approaches (**Fig. 1** upper path), and the alternative ‘individual correlation’-based approach we introduce here (**Fig. 1** lower path). Data shown is from a study of gene expression in responders and nonresponders to interferon/ribavirin therapy to Hepatitis C [25]. The strong pairwise correlation within each individual is lost in the average-based approach, whereas it is clearly exposed by the individual correlation-based approach.

To systematically examine the differences between group average- and individual correlation-based approaches, we evaluated them using a biomarker discovery scenario across four different published datasets (**Table 1**). Additionally, we evaluated an alternative strategy of concatenating the time series measurements across individuals within the groups, as applied earlier [26], [27]. More specifically, we considered here six different approaches: (i) conventional differential expression analysis using the Bioconductor *limma* package [28]; (ii) differential co-expression analysis after group averaging using the popular WGCNA approach or (iii) the more recent DCp method; (iv) differential co-expression analysis after concatenating the time series within the groups using WGCNA; (v) differential co-expression analysis on individual correlations using WGCNA or (vi) an alternative approach we introduce here, which we name Dynamically Co-expressed Neighborhoods (DCeN), that takes into account also the neighbor-wise co-expression changes instead of a simple total connectivity of a gene. Each method was used to rank all the measured genes, with the idea that genes that have an important functional role in the transcriptional response should appear consistently among the top-ranked genes.

**Table 1:** Time series gene expression datasets used in this study.

Dataset	Study organism	Array type	Individuals/strains	Sample series	GEO/Array Express accession number	Reference
*HCV*	Human	Affymetrix Human Genome U133A	17 responders, 13 nonresponders	0, 1, 2, 7,14, 28 days	GSE7123	[25]
*LPS*	Mouse	Affymetrix Mouse Genome 430A 2.0	2 mice stimulated with LPS, 1 non-stimulated mouse	0.5, 1, 2, 4, 6, 8, 12, 16, 24 hours	GSE17721	[29]
*CDC13*	Yeast	Affymetrix Yeast Genome 2.0	3 wild-type yeast strains, 3 strains carrying *cdc13-1* mutation	0, 1, 2, 3, 4 hours	E-MEXP-1551	[30]
*Th2*	Human	Affymetrix Human Genome U133 Plus 2.0	3 Th2-polarized, 3 non-Th2-polarized	Thp, 0.5, 1, 2, 4, 6, 12, 24, 48, 72 hours	GSE18017	[32]

## Results

### Consistency

**Figure 2:**
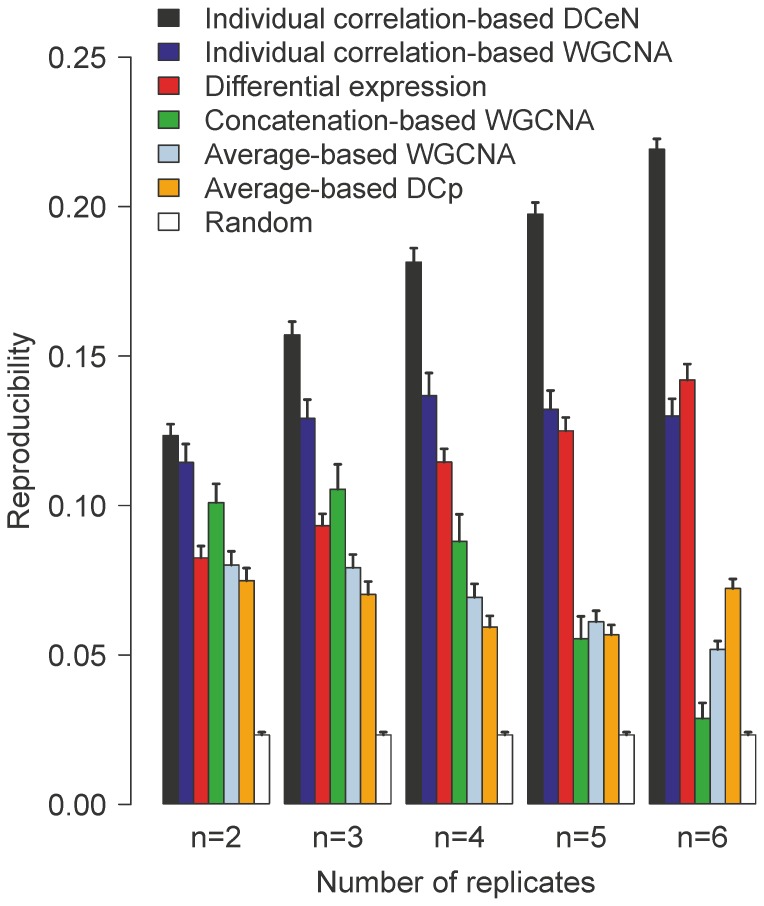
Reproducibility of detection in independent subsamples of human hepatitis C virus (*HCV*) dataset among different methods. Pairs of independent sub-datasets of two to six replicates (*x*-axis) were generated by random sampling without replacement. Reproducibility is here defined as the overlap of the top-ranked detections at various top list sizes. For summary, the average relative reproducibility over 100 pairs of independent sub-datasets is shown at top list size of 200 (*y*-axis). Error bars show the empirical standard error of the mean. Complete reproducibility values are provided in **Figure S1**. Each gene ranking method was applied to identical data sets. (DCeN, Differential Co-expression Networks; WGCNA, Weighted Gene Co-expression Network Analysis; DCp, Differential Co-expression profiles; Random, random permutation).

We assessed the reproducibility of the top-ranked genes in the human hepatitis C virus (HCV) treatment dataset [25] across independent subsamples of two to six replicates (**Fig. 2** and **Fig. S1**). Throughout all numbers of replicates tested, the reproducibility of our DCeN method was significantly higher than that of the current state-of-the-art gene ranking methods based on differential expression or differential co-expression (**Fig. 2**; Wilcoxon signed rank test, *p*<0.01). In general, the individual correlation-based approach improved markedly also the performance of WGCNA when compared to the previously applied average-based and concatenation-based approaches, although its reproducibility remained significantly lower than that of DCeN. We also tested whether subtracting the gene-wise average intensity from each individual before concatenation would improve the concatenation-based approach but we did not observe any significant effect (**Fig. S2**; Wilcoxon signed rank test, *p*>0.05). Not unexpectedly, reproducibility increased with the number of replicates for the DCeN and the differential expression method. However, for the previously proposed differential co-expression methods WGCNA and DCp, such increase was not evident.

### Overall biological relevance

To systematically assess the overall biological relevance of each computational method, we applied them to two publicly available gene expression time series datasets in which the biological importance of many genes had been independently assessed using RNA interference (RNAi) or gene deletions.

**Figure 3:**
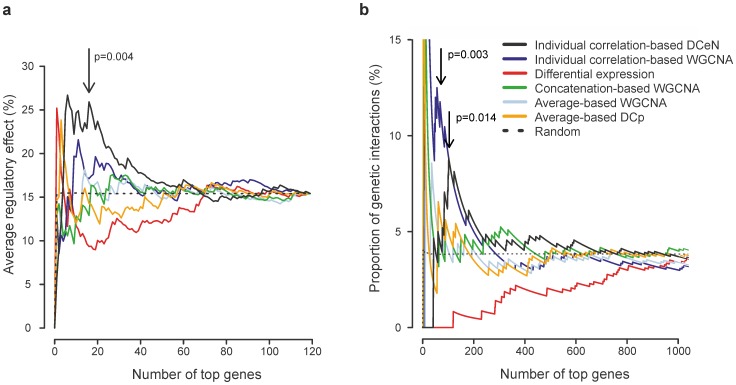
Biological relevance of results from DCeN and current state-of-the-art gene ranking methods. **(a)** In the *LPS* data set, average regulatory effect of the top candidates (*y*-axis) was investigated as a function of the top list size (*x*-axis). Here, the regulatory effect of any validated transcription factors in the *LPS* data set was measured as the percentage of signature genes identified as its targets. Statistical significance was assessed using permutation tests. The most significant *p-*value is indicated by an arrow. **(b)** In the *CDC13* data set, biological relevance of the top candidates was measured as the proportion of the top candidates showing genetic interaction with the *cdc13-1* mutation (*y*-axis) as a function of the top list size (*x*-axis). Statistical significance was assessed by permutation. The most significant *p*-values are indicated by an arrow. (DCeN, Differential Co-expression Networks; WGCNA, Weighted Gene Co-expression Network Analysis; DCp, Differential Co-expression profiles; Random, random permutation).

In the *LPS* data set, the transcriptome of mouse dendritic cells was profiled at multiple time points after stimulation with various toll-like receptor ligands, and without stimulation. Independently, the biological role of 125 transcription factors had been probed and validated using RNAi and a set of signature genes [29]. We used each computational analysis method to rank the validated factors, and then quantified an average regulatory effect of the top-ranked factors on the basis of the RNAi data; the regulatory effect of a factor was defined as the percentage of the signature genes that were identified as its targets (**Fig. 3a**). Genes with the highest DCeN values tended to have, on average, larger regulatory effects than expected by chance (permutation test, most significant *p* = 0.004). Notably, no such effect could be observed when the other methods were used (permutation test, *p*>0.1 at all top list sizes).

In the *CDC13* data set, time series of gene expression profiles in wild-type yeast strains and in strains carrying the temperature-sensitive cdc13-1 mutation were acquired after a temperature change [30]. Additionally, independent genome-wide screens for deletion mutants showing genetic interactions with the cdc13-1 mutation had been performed [30], [31]. Ranking the screened genes on the basis of their temporal gene expression revealed that the genes with the highest DCeN values were enriched with genetic interactions with cdc13-1 (permutation test, most significant *p* = 0.014; **Fig. 3b**). In these data, also the individual correlation-based WGCNA produced significant enrichment (permutation test, most significant *p* = 0.003). Again, no statistically significant enrichment was observed for the other methods (permutation test, *p*>0.05 at all top list sizes from 1 to 1000).

Overall, the traditional differential expression or differential co-expression measures performed surprisingly poorly, whereas the individual correlation-based approaches improved the detections. Only DCeN identified significant numbers of functionally relevant genes in both datasets, while individual correlation-based WGCNA performed well only in the *CDC13* data.

### Detection of specific co-expression patterns

Finally, we performed a detailed manual analysis using DCeN on the human T helper 2 (*Th2*) cell differentiation dataset [32]. Human activated CD4+ T cells were profiled with and without polarization towards Th2 with IL-4. Among the 115 top-ranked genes for differential co-expression (permutation test, *p*<0.05) were several factors with previously assigned functions specific to Th2 cells, such as ITK, ICOS, LEF1, and GATA3, a well-known master regulator of Th2 cell differentiation, as well as genes that have only more recently been proposed to play an important role in Th2 cell differentiation, such as SOCS2 and STAT3 [33]–[38]. Also, the strong enrichment of the DCeN genes for immune response (19%, false discovery rate FDR<10^−5^) and regulation of apoptosis (18%, FDR<10^−4^) was consistent with the different Th cell subsets playing a critical role in immune response and having different susceptibility to apoptosis.

Interestingly, there were eleven transcription regulators (ARID5B, EPAS1, GATA3, IRF1, IRF7, IRF9, LEF1, SP100, STAT3, TRIM22, XBP1) among the detected genes, seven of which (64%; EPAS1, IRF1, IRF7, IRF9, LEF1, STAT3, XBP1) corresponded to transcription factor motifs recently identified as enriched in lineage-specific enhancers compared to random locations in genome (*p*<0.05), suggesting their regulatory role in T cell polarization [39]. This was significantly more than expected by chance (Fisher exact test *p*<0.01). Notably, two of these factors, IRF9 and LEF1, would have been completely missed in the present study on the basis of differential expression only. We note in passing that 20% of our detections did not show evidence for differential expression [32].

**Figure 4:**
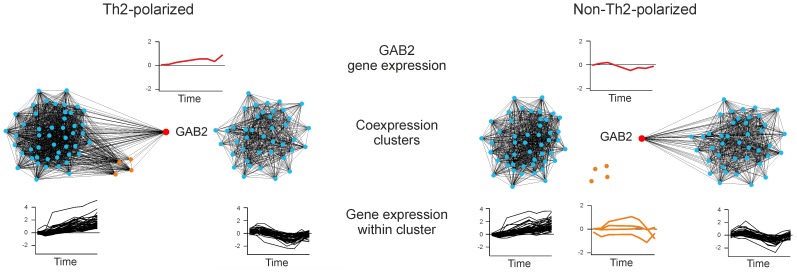
Switching pattern for GAB2 in human T helper 2 cell differentiation (*Th2*) dataset, detected by DCeN. Co-expression clusters, visualized here using Cytoscape, contain those genes with the largest difference in co-expression with GAB2 between the two experiments (top 0.5%). Connecting edges indicate strong correlation (co-expression weight>0.95). Co-expression of GAB2 with other genes switches between different co-expression clusters, depending on whether polarization towards Th2 was induced or not.

Further detailed analysis revealed patterns difficult to extract using existing approaches. One example is a ‘switching pattern’ exhibited by GAB2 (**Fig. 4**) whose expression changes between co-expression with one cluster of consistently co-expressed genes under Th2 polarizing conditions to another consistently co-expressed cluster under non-polarizing conditions. GAB2 is an adaptor protein that activates PI3K and Akt, which subsequently regulates IL-4 production [40]. Recently, GAB2 was suggested to be a potential key player in the IL-4-STAT6 regulatory feedback loop [39]. Notably, while most of the genes in the co-expression clusters remained consistently co-expressed with each other under both conditions, we identified also four other genes (GATA3, KPNA6, PPP1R14A, RRS1) that were part of the same co-expression cluster as GAB2 under the Th2 polarizing conditions but not under the non-polarizing conditions. Of these, GATA3 and RRS1 were identified in our DCeN analysis, whereas KPNA6 and PPP1R14A fell slightly below our significance thresholds (DCeN ranks 148 and 127, respectively).

## Discussion

Our results suggest surprising significant weaknesses of state-of-the-art data analysis approaches to detect differential co-expression from grouped time-resolved transcriptomic data. In our evaluation across the few published datasets of this type, the DCeN method, which we introduced here, was the only approach to consistently extract significant numbers of genes corroborated in independent studies, and highlighted dynamic association of known immune regulators under corresponding experimental conditions. The insights enabled by the global measurement of dynamic patterns across groups of individuals will make this type of data increasingly attractive, and will certainly create a broader basis for studies such as this in the future.

Nonetheless, the observed difference in performance is striking. The DCeN method itself is neither particularly elaborate, nor adapted to the particular data sets used here, suggesting that the best explanation of the observed performance difference is its fundamentally different analysis approach based on individual correlation instead of group averages. Computing correlation between pairs of genes within each individual is unaffected by variability between individuals, whereas the averaging step in state-of-the-art approaches potentially loses a significant amount of information, particularly across individuals, or through other aspects that are typically hard to control.

This interpretation of our results has different corollaries for experiment design and analysis, in particular, in the case of transcriptomic measurements across individuals. Firstly, the apparent importance of robustness against variation, which is likely due to – typically a priori unknown, but potentially widespread – variations in speed and timing, underlines the necessity to acquire transcriptome profiles at multiple time points to ensure that characteristic changes are captured in each time course. Secondly, clustering methods, as close relatives to co-expression detection methods, need to take the potentially different timing between individuals into account as well. Thirdly, more detailed computational models of biological processes may have to better accommodate heterogeneity, for instance, by explicitly modeling heterogeneous speeds of low- and high-level biological processes, to enable satisfactory agreement with experimental data and to attain the high level of robust predictive performance required for their broad use in biomedical applications.

## Materials and Methods

### Dynamically Co-Expressed Neighborhoods (DCeN)

**Figure 5:**
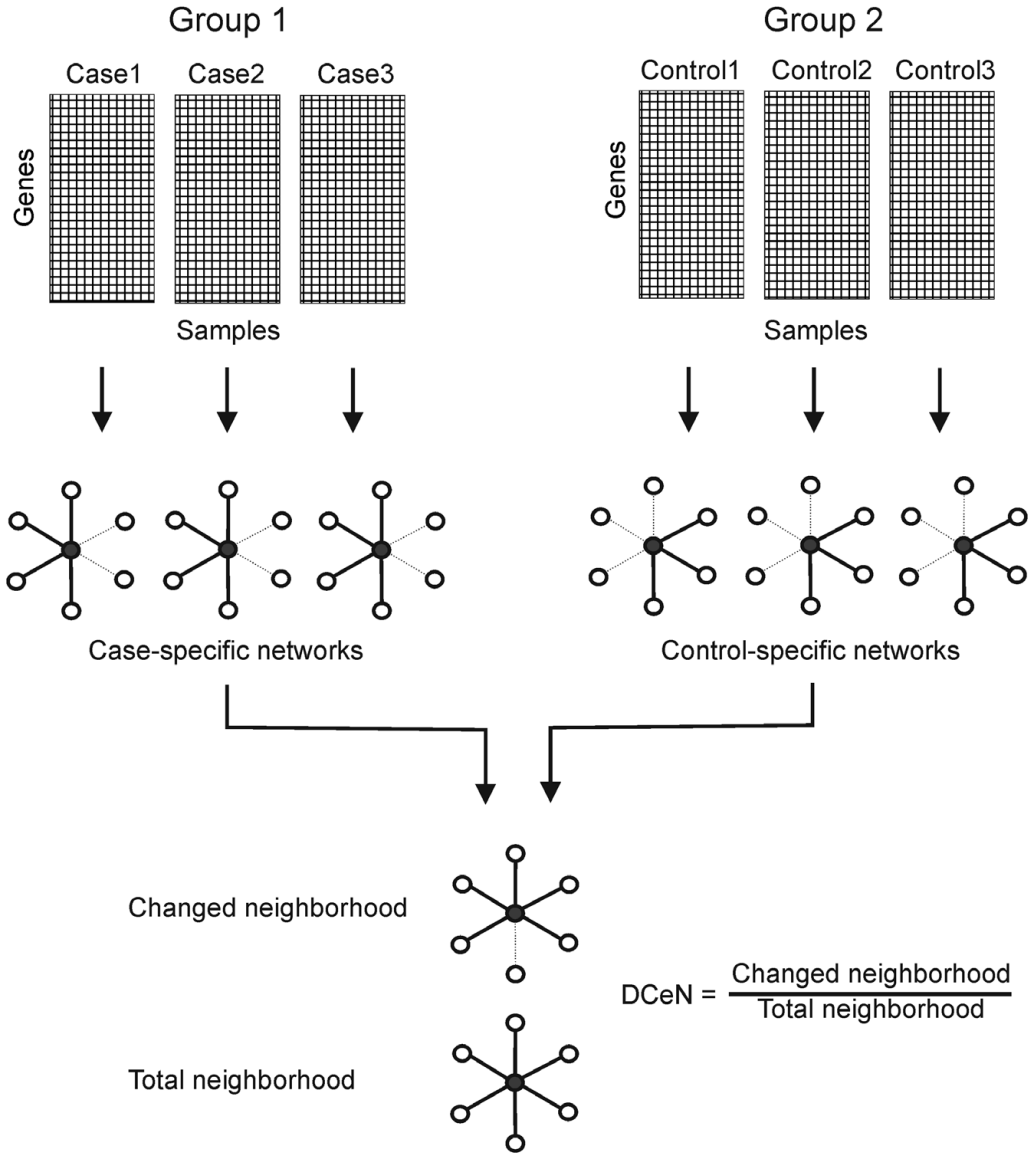
Schematic illustration of Dynamically Co-Expressed Neighborhoods (DCeN). Time series data is used to construct a separate gene co-expression network for each individual case and control. For a particular gene (the gray node), only those neighbors are retained that are consistently co-expressed across the replicates in at least one group (solid edges). These neighbors are then used to determine the changed and total neighborhoods of the gene. The changed neighborhood corresponds to the cumulative difference in the co-expression values of the gene between the two groups. The total neighborhood corresponds to the total connectivity of the gene across the groups. Finally, DCeN is defined as the proportion of the total neighborhood that is changed. See Materials and Methods for details of the procedure.

A schematic illustration of the general DCeN procedure is shown in **Figure 5**. Given two sets of gene expression profiles and a gene *g*, the DCeN method assigns to *g* a score *d_g_* that quantifies the differences in the co-expression of *g* with any other gene between the two sets. In the present study, we determine the score *d_g_* based on the Pearson correlation *r_ghi_* between genes *g* and *h* for an individual *i*, after taking the number *m* of time points into account.

Specifically, let *p_ghi_* be the significance of an observed correlation *r_ghi_*, determined using the statistic 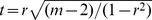, which has a Student's *t*-distribution with 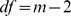 degrees of freedom under the hypothesis of no correlation [41]. A weighted graph for individual *i* is then constructed using the weights 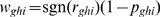
for each pair 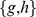 of genes, where sgn is the sign function. Finally, to focus on gene-gene correlations that are consistent across individuals, only gene pairs 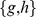 with a positive or negative correlation with *p_ghi_*<0.05 in at least 25% of the individuals *i* in at least one group are used for further analysis (with a minimum of two individuals if available). The rather liberal significance threshold was selected to discard only those links that are most likely to be uninteresting. Changing the threshold to 0.01 or 0.25 did not have a large impact on results but all the thresholds improved markedly the reproducibility as compared to analyses without any prefiltering (**Fig. S3**). We use *a_gh_* to denote whether the relationship between genes *g* and *h* satisfies the above criterion (*a_gh_* = 1) or not (*a_gh_* = 0). Dynamically Co-expressed Neighborhoods (DCeN) are finally determined using averaged weights 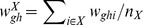 and 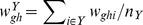, and the formula 
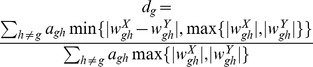
, if 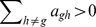.

Here, *n_X_* and *n_Y_* are the numbers of individuals in the two groups *X* and *Y* under comparison. The numerator corresponds to the cumulative difference in the neighborhood of a gene *g* between the two groups (changed neighborhood), whereas the denominator reflects the total connectivity of gene *g* across the groups (total neighborhood). If the neighborhoods are equal under both conditions, then *d_g_* = 0. The maximum value of 1 is obtained when the stronger (positive or negative) weight over the conditions is zero or of opposite sign under the other condition. If 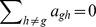, we define *d_g_* = 0.

### DE, WGCNA, and DCp

Differential expression (DE) was determined using the Bioconductor *limma* package [28]. Genes were ranked according to the moderated *F*-test. Weighted Gene Co-expression Network Analysis (WGCNA) [42] and Differential Co-expression Profile analysis (DCp) [22] were applied using the R package DCGL following the instructions provided in the package manual [43]. DCp was recently suggested to improve the detection of differentially co-expressed genes, whereas WGCNA showed the best performance among the popular existing algorithms in the same study [22].

### Datasets used for evaluation

The human hepatitis C virus (*HCV*) dataset [25] used for the reproducibility analysis included gene expression profiles of peripheral blood mononuclear cells from Caucasian American patients with chronic HCV infection. Gene expression was profiled at six time points after initiation of treatment. The preprocessed data on 30 patients with measurements at each time point was downloaded [44], including 17 patients showing good (marked or intermediate) response and 13 patients showing poor response.

The mouse lipopolysaccharide (*LPS*) dataset [29] includes gene expression profiles of mouse dendritic cells exposed to LPS. Gene expression was profiled in duplicate series at nine time points after stimulation with LPS, and in a non-stimulated control series. The normalized data was downloaded from GEO (GSE17721). A set of 125 candidate regulators were validated in the original study using RNAi and a set of signature genes [29]. In the present study, the regulatory effect of a candidate regulator was defined as the percentage of the signature genes that were identified as its targets at 95% confidence in terms of both gene-specific and perturbation-specific noise.

The yeast *CDC13* dataset [30] includes gene expression profiles of three wild-type yeast strains and three strains carrying the temperature sensitive *cdc13-1* mutation. Gene expression was profiled initially at 23°C and then at additional four time points after a temperature shift to 30°C. The data was preprocessed as described earlier [45]. In two separate studies, the same laboratory carried out genome-wide screens for deletion mutants showing genetic interactions with the *cdc13-1* mutation [31], [46]. Here, we focused on the interactions that were reproducibly identified in both of the screens.

The human T helper 2 (*Th2*) cell differentiation dataset [32] includes gene expression profiles of human CD4+ T cells activated (Th0), or activated and polarized towards Th2 with IL-4. Gene expression was profiled in triplicate series in naïve Th precursor (Thp) cells and at nine time points after activation and initiation of polarization. Here, we used those seven time points that had gene expression data from each replicate. The quantile-normalized probe-level data was transformed into probe set-level signal log-ratios between each non-Thp sample and the corresponding Thp sample using the probe-level expression change averaging procedure [47], as described in GEO (GSE18017).

From the original preprocessed datasets, only those probe sets were retained that mapped to a unique Entrez ID. If multiple probe sets mapped to the same Entrez ID then the one with the highest overall intensity was selected [48]. In the LPS and CDC13 datasets, we focused on the genes tested in the independent RNAi or gene deletion experiments. In the HCV and Th2 datasets, a non-specific filter was applied to focus on the top 20% of the genes with the highest overall variance [49].

### Statistical analyses

When evaluating the statistical significance of the average regulatory effects or the enrichment for genetic interactions among the top-ranked genes, the observed values were compared to those obtained when repeating the same analysis 10000 times after randomly permuting the gene labels. Statistical significance of the DCeN detections was estimated using the permutation approach introduced earlier [22]. The sample labels were randomly permuted 100 times to form an empirical null distribution.

### Implementation

All differential expression and differential co-expression analyses as well as the statistical analyses were implemented using the R statistical software (http://www.r-project.org/). An R package implementing the DCeN method is freely available under the terms of the GNU General Public License version 3 or newer on our website (http://www.btk.fi/research/research-groups/elo/). The networks were visualized using Cytoscape [50].

## Supporting Information

Figure S1 **Reproducibility of detections in independent subsamples of the human hepatitis C virus (HCV) dataset.** The performance of the Dynamically Co-expressed Neighborhoods (DCeN) method was compared to that of the current state-of-the-art gene ranking methods using differential expression (DE) or differential co-expression (WGCNA and DCp). Pairs of independent subdatasets were generated by randomly sampling *n* = 2, …,6 cases from the groups of 17 responders and 13 nonresponders without replacement. Reproducibility was defined as the overlap of the top-ranked detections at various top list sizes. Average reproducibility over 100 pairs of datasets (*y*-axis) is shown as a function of the top list size (*x*-axis). The same datasets were analyzed with each gene ranking method.
(TIF)

Figure S2 **Effect of subtracting the gene-wise average of each individual on the reproducibility of the concatenation-based approach.** Pairs of independent subdatasets were generated by randomly sampling *n* = 2, …,6 cases from the groups of 17 responders and 13 nonresponders without replacement. Reproducibility was defined as the overlap of the top-ranked detections at various top list sizes. For summary, reproducibility of 100 pairs of datasets is shown at top list size of 200 (*y*-axis). The same datasets were analyzed with each gene ranking method. The boxes show the median and the interquartile range (IQR) of the observed reproducibility, the whiskers indicate their range and the points correspond to extreme observations with values greater than 1.5 times the IQR. The difference between the approaches was not significant at any sample size (Wilcoxon signed rank test, *p*>0.05).
(TIF)

Figure S3 **Effect of the prefiltering threshold on the reproducibility of the Dynamically Co-expressed Neighborhoods (DCeN) method.** Reproducibility was assessed in the human hepatitis C virus (HCV) data. Pairs of independent subdatasets were generated by randomly sampling five cases from the groups of 17 responders and 13 nonresponders without replacement. Reproducibility was defined as the overlap of the top-ranked detections at various top list sizes. Average reproducibility over 10 pairs of datasets (*y*-axis) is shown as a function of the top list size (*x*-axis). The same datasets were analyzed with each prefiltering threshold or without any prefiltering. See the Materials and Methods section for details of the prefiltering threshold.
(TIF)
