# Supplementary figures and images for: Analysis of Time-Resolved Gene Expression Measurements across Individuals
Source: PLoS One. 2013 Dec 9;8(12):e82340. doi: 10.1371/journal.pone.0082340 (PMC3857324; doi:10.1371/journal.pone.0082340)

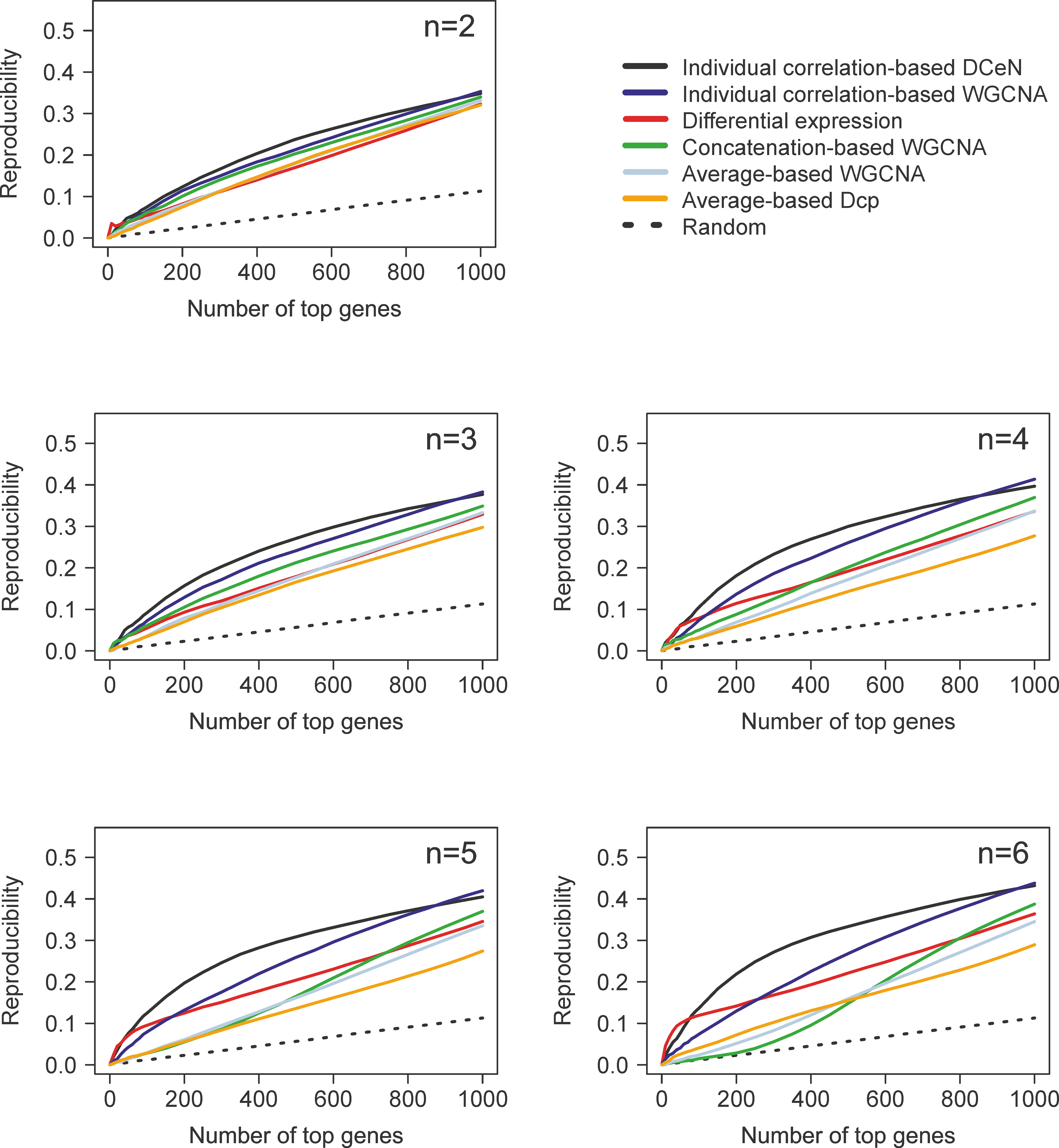

Supplement: Figure S1 — Reproducibility of detections in independent subsamples of the human hepatitis C virus (HCV) dataset. The performance of the Dynamically Co-expressed Neighborhoods (DCeN) method was compared to that of the current state-of-the-art gene ranking methods using differential expression (DE) or differential co-expression (WGCNA and DCp). Pairs of independent subdatasets were generated by randomly sampling n = 2, …,6 cases from the groups of 17 responders and 13 nonresponders without replacement. Reproducibility was defined as the overlap of the top-ranked detections at various top list sizes. Average reproducibility over 100 pairs of datasets (y-axis) is shown as a function of the top list size (x-axis). The same datasets were analyzed with each gene ranking method. (TIF) [file pone.0082340.s001.tif]

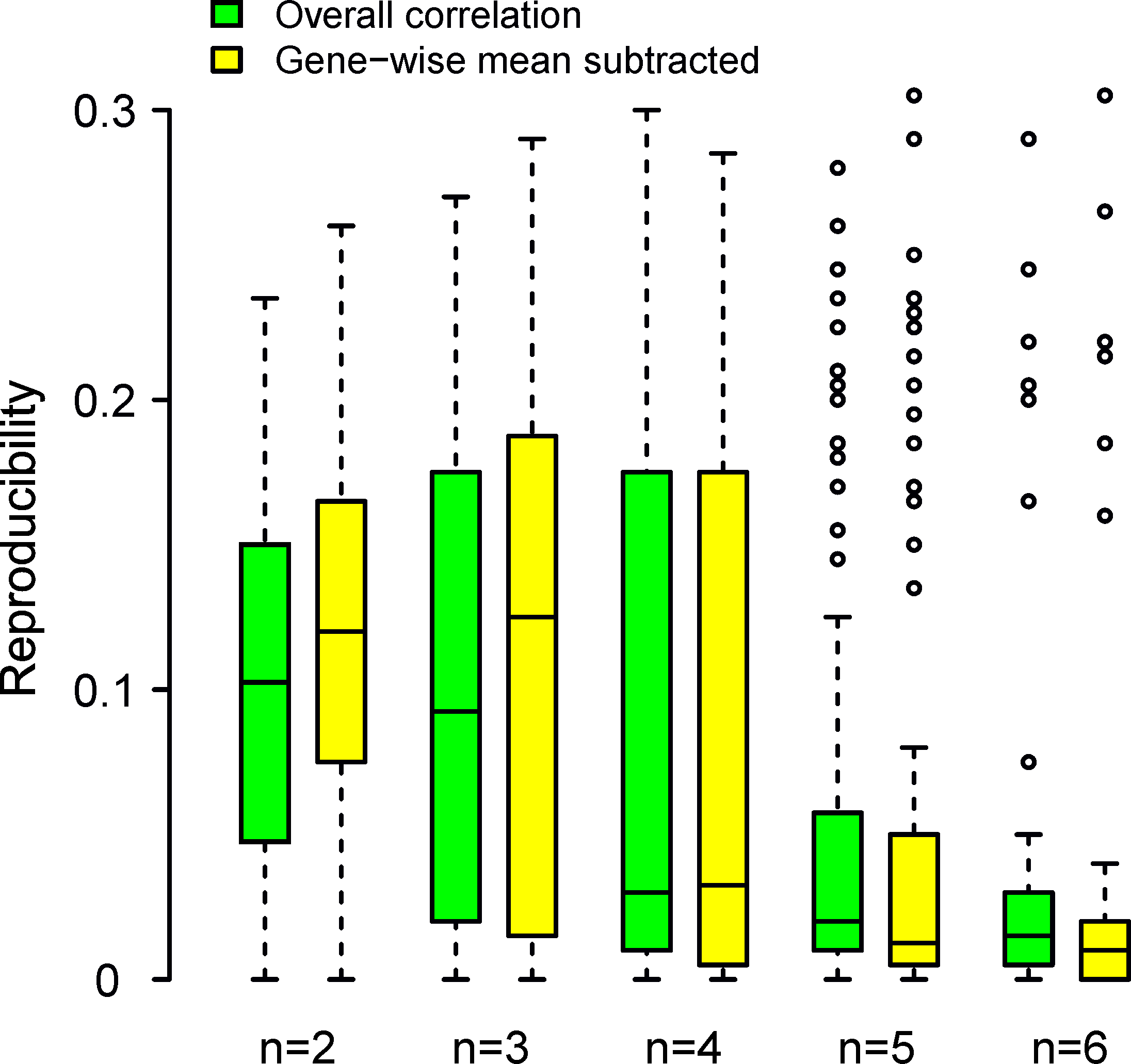

Supplement: Figure S2 — Effect of subtracting the gene-wise average of each individual on the reproducibility of the concatenation-based approach. Pairs of independent subdatasets were generated by randomly sampling n = 2, …,6 cases from the groups of 17 responders and 13 nonresponders without replacement. Reproducibility was defined as the overlap of the top-ranked detections at various top list sizes. For summary, reproducibility of 100 pairs of datasets is shown at top list size of 200 (y-axis). The same datasets were analyzed with each gene ranking method. The boxes show the median and the interquartile range (IQR) of the observed reproducibility, the whiskers indicate their range and the points correspond to extreme observations with values greater than 1.5 times the IQR. The difference between the approaches was not significant at any sample size (Wilcoxon signed rank test, p>0.05). (TIF) [file pone.0082340.s002.tif]

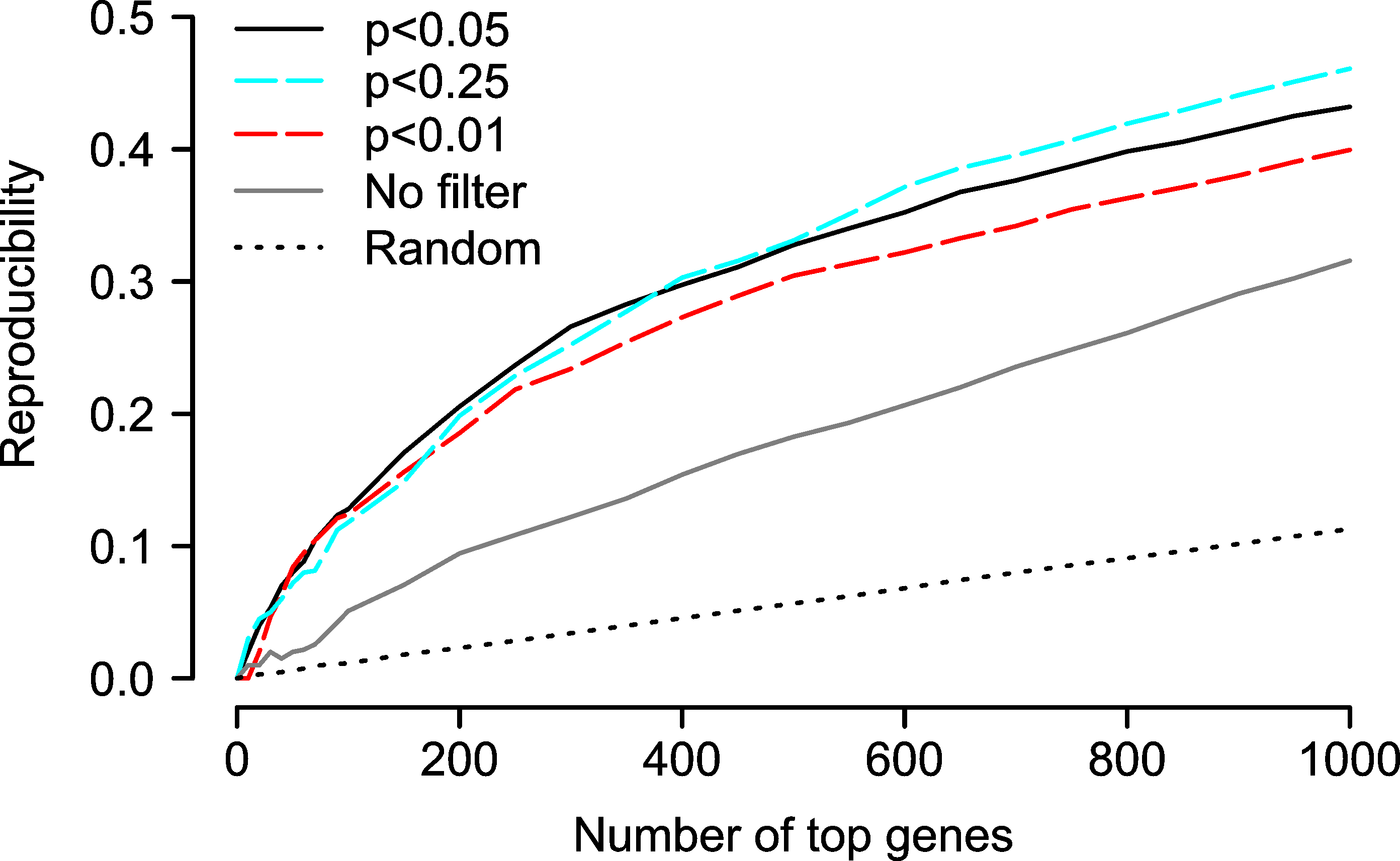

Supplement: Figure S3 — Effect of the prefiltering threshold on the reproducibility of the Dynamically Co-expressed Neighborhoods (DCeN) method. Reproducibility was assessed in the human hepatitis C virus (HCV) data. Pairs of independent subdatasets were generated by randomly sampling five cases from the groups of 17 responders and 13 nonresponders without replacement. Reproducibility was defined as the overlap of the top-ranked detections at various top list sizes. Average reproducibility over 10 pairs of datasets (y-axis) is shown as a function of the top list size (x-axis). The same datasets were analyzed with each prefiltering threshold or without any prefiltering. See the Materials and Methods section for details of the prefiltering threshold. (TIF) [file pone.0082340.s003.tif]
